# Phytoplankton Cell Lysis Associated with Polyunsaturated Aldehyde Release in the Northern Adriatic Sea

**DOI:** 10.1371/journal.pone.0085947

**Published:** 2014-01-31

**Authors:** François Ribalet, Mauro Bastianini, Charles Vidoudez, Francesco Acri, John Berges, Adrianna Ianora, Antonio Miralto, Georg Pohnert, Giovanna Romano, Thomas Wichard, Raffaella Casotti

**Affiliations:** 1 Stazione Zoologica Anton Dohrn di Napoli, Napoli, Italy; 2 ISMAR CNR Istituto Scienze Marine, Venezia, Italy; 3 Friedrich Schiller University Jena, Institute for Inorganic and Analytical Chemistry, Jena School for Microbial Communication, Jena, Germany; 4 Department of Biological Sciences and School of Freshwater Sciences, University of Wisconsin-Milwaukee, Milwaukee, Wisconsin, United States of America; University of New South Wales, Australia

## Abstract

Diatoms are able to react to biotic and abiotic stress, such as competition, predation and unfavorable growth conditions, by producing bioactive compounds including polyunsaturated aldehydes (PUAs). PUAs have been shown to act against grazers and either enhance or inhibit the growth of different phytoplankton and bacteria both in culture and in the field. Presence of nanomolar concentrations of dissolved PUAs in seawater has been reported in the North Adriatic Sea (Mediterranean), suggesting that these compounds are released in seawater following diatom cell lysis. However, the origin of the PUAs and their effects on natural phytoplankton assemblages remain unclear. Here we present data from four oceanographic cruises that took place during diatom blooms in the northern Adriatic Sea where concentrations of particulate and dissolved PUAs were monitored along with phytoplankton cell lysis. Cell lysis was positively correlated with both concentrations of particulate and dissolved PUAs (R = 0.69 and R = 0.77, respectively), supporting the hypothesis that these compounds are released by cell lysis. However, the highest concentration of dissolved PUAs (2.53 nM) was measured when cell lysis was high (0.24 d^−1^) but no known PUA-producing diatoms were detected, suggesting either that other organisms can produce PUAs or that PUA-producing enzymes retain activity extracellularly after diatom cells have lysed. Although *in situ* concentrations of dissolved PUAs were one to three orders of magnitude lower than those typically used in laboratory culture experiments, we argue that concentrations produced in the field could induce similar effects to those observed in culture and therefore may help shape plankton community composition and function in the oceans.

## Introduction

Diatoms are ubiquitous organisms, responsible for about one fifth of the photosynthesis on Earth [1]. They have developed sophisticated regulatory mechanisms to perceive changes in environmental conditions and respond accordingly [2]. Some of these mechanisms rely upon the production of bioactive molecules, the most well-known being the polyunsaturated fatty acid derivatives, called oxylipins [3]–[5].

In higher plants, oxylipins play an important role in acclimation to environmental stresses due to wounding, grazing by predators or competition for limiting nutrients [6] and can trigger several physiological responses, ranging from inducing cell division to cell lysis depending on the levels of oxylipins produced [7]. Similar to plants [8], diatom oxylipins such as polyunsaturated aldehydes (PUAs) are associated with a chemical defense system that impairs the reproductive success of copepods and other invertebrates [3], [5], inhibits the growth of cultured phytoplankton [9], [10] and affects growth in bacteria communities both in culture [11], [12] and in the field [13].

Diatoms produce PUAs in at least three steps. First, chloroplast-membrane-localized glycolipids and plasma-membrane-localized phospholipids are hydrolyzed to generate free polyunsaturated fatty acids (PUFAs) [14], [15]. Then, the free PUFAs are acted on by lipoxygenases that generate hydroxyperoxy fatty acids. Finally, hydropeoxy fatty acids are transformed into PUAs [16], [17]. In contrast to plants, diatom-derived PUAs are almost never found in intact cells but are synthesized mainly after membrane disruption [18]. PUA production takes place as long as enzymes are in contact with precursor free fatty acids and is not inhibited by the amount of PUAs produced [19].

Diatom PUAs appear to be part of a nitric-oxide-based stress surveillance system that mediates intracellular communication involved in regulating stress response to unfavorable environmental conditions [4]. PUA production and release in the water remains low under optimal growth conditions, but increases as conditions become poorer, e.g., nutrients become limiting [20], [21]. Such a surveillance mechanism depends upon the amount of PUA produced. High PUA exposure triggers cell lysis in exponentially-growing diatoms while lower exposures induce resistance, leading to decreasing effects in successive exposures [4]. In contrast, PUAs can accelerate cell lysis in diatoms in the late stationary phase of growth, even if they have been exposed to PUAs previously [22]. In nature, this surveillance mechanism would confer an advantage to PUA-producing diatoms at the peak of the bloom, when nutrient conditions progressively become limiting, by inhibiting the growth of competitors. Later on, when resources become depleted and cells begin to lyse, large PUAs releases could induce a synchronized cell lysis, leading to the sharp declines of diatoms commonly observed in nature [23].

The relevance of culture experiments has been questioned because PUA concentrations are typically orders of magnitude higher than those predicted in the field [24]. However, Dittami et al. [25] have shown that repeated application of low doses (20 nM) of octadienal caused cell lysis of a non PUA-producing *Thalassiosira rotula* strain (CCMP 1018), a concentration that is in the same range as that reported by Vidoudez et al. [26] in surface water during blooms of the diatom *Skeletonema marinoi* in the northern Adriatic Sea (Mediterranean). At present our understanding of the dynamics of PUA releases and effects on natural phytoplankton assemblages is very poor.

Here we present data from an extended field survey of PUAs in the northern Adriatic Sea (Mediterranean) that was carried out during March 2002, 2004, 2005 and 2006. We measured phytoplankton cell lysis along with the concentrations of PUAs produced after chemical or mechanical disruption of the cells (hereafter termed “particulate PUAs”) as well as dissolved PUAs present in seawater. Our results show that cell lysis is positively correlated with particulate and dissolved concentrations of PUAs, supporting the hypothesis that these compounds are released in seawater following cell lysis.

## Methods

**Figure 1:**
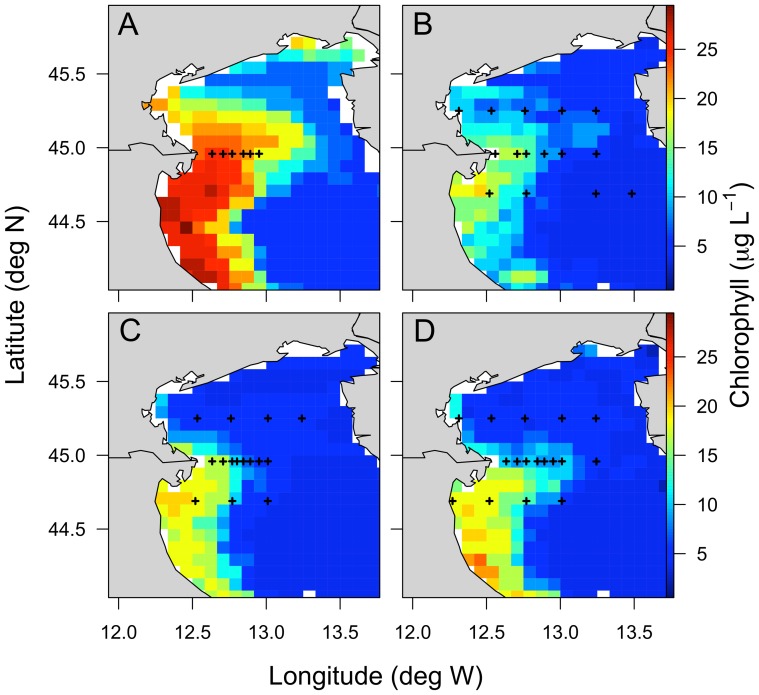
Chlorophyll *a* distribution and station location in the North Adriatic Sea. Chlorophyll *a* (μg L^−1^) was determined from SeaWifs datasets (http://gdata1.sci.gsfc.nasa.gov/). Sampling stations are indicaed with black crossses along three transects, transect 1 (north), transect 2 and transect 3 (south). Transect were sampled in March a) 2002, b) 2004, c) 2005 and d) 2006.

Four oceanographic cruises were conducted in the North Adriatic Sea in March 2002, 2004, 2005 and 2006 aboard the *RV Urania* and *RV Dallaporta*. Samples were taken at the surface using 5 or 10-L Niskin bottles from stations located along three transects (Fig. 1). Only transect 2, located at the Po river mouth, was sampled in March 2002. In March 2005 and 2006, two coastal stations were sampled every 3 h for 27h in order to investigate the dynamics of PUA production and cell lysis over a diel cycle. Permission to operate at the sampling stations was given by the Harbour Master Stations (Capitanerie di Porto) of Venice and Ravenna, Italy. The work did not involve any endangered or protected biological species.

### Oceanographic data

Dissolved inorganic nutrients (phosphate, silicic acid, nitrite and nitrate) were analyzed by colorimetry with an autoanalyzer (Systea-Alliance) according to Grasshoff et al. (1999)[27]. Because nitrite concentrations were always very low (<0.3 μM), dissolved inorganic nitrogen (DIN) concentrations were expressed as the sum of the nitrite and nitrate. Samples for particulate C and N (POC and PON, respectively) were filtered on acidified pre-combusted (450°C, 24 h) GF/F filters and stored at −80°C until analysis with a CHN elemental analyzer (Perkin Elmer 2400). Phytoplankton cell numbers and species composition were determined on fixed samples (buffered formaldehyde 2%) after concentration by sedimentation [28], using an inverted microscope (Zeiss Axiovert 35). Samples for chlorophyll *a* analysis were obtained by gentle filtration (<100 mm Hg) onto GF/F filters and storage at −80°C until further analysis. The filters were ground in 100% methanol and the extract injected into a Beckman System Gold HPLC. Monthly averages of satellite-determined chlorophyll *a* distribution were obtained from SeaWIFS data (http://gdata1.sci.gsfc.nasa.gov/).

### Measurements of cell lysis

Phytoplankton lysis rates were estimated following an improved version [29] of the esterase method [30]. The method is based on the assumption that esterases are strictly intracellular enzymes that are released only by cell lysis; esterase activity is measured as increased fluorescence due to cleavage of fluorescein diacetate (FDA). Dissolved esterase activity (DEA) was measured in 0.22 µm filtrates (Steriflip-GP filter Unit, Millipore, Milano, IT) and particulate esterase activity (PEA) was measured on untreated samples, after correction for non-enzymatic hydrolysis of FDA (measured after removing all esterases from the sample using a <10 kDa centrifuge filter (Amicon Ultra-4 PLGC Ultracel-PL Membrane, Millipore, IT, Milan). Decay rates of DEA were measured in each sample at t = 0 (performed no later than 30 minutes after water collection) and after 24 h incubation of the 0.22 µm filtrate in the dark at *in-situ* temperature (maintained by thermostat baths). All measurements were performed under standardized conditions: 0.1 mL of Tris–HCl (pH 8.0; final concentrations 0.5 mM) with EDTA (final concentrations 0.5 mM) and 20 µl of FDA (FDA, Sigma-Aldrich Inc., Milano, IT, diluted in acetone; final concentration 20 µM) were added at time *t* = 0 into 1.9 mL of sample. The increase in fluorescence of the samples was recorded (excitation  = 490 nm, emission  = 520 nm) following an incubation at 20°C for 60 min using a CaryEclipse (Varian) fluorometer. Fluorescence was converted into fluorescein concentration using an internal standard (Fluorescein, Sigma-Aldrich Inc., Milano, IT) that was added (final concentration 5 nM) immediately to the sample after the measurement at time *t* = 60 min. Esterase activities were measured in three replicates (coefficient of variation averaged 5%). The cell lysis rate was calculated as the decrease in PEA with time due to the production of DEA during cell lysis (produced on an hourly basis) using the following equations [29]:
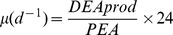
where 
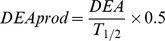
 and *T*_1/2_ represents the DEA turnover time, calculated from the esterase decay rate measured in each sample.

### *In situ* quantification of PUAs

Since PUAs are particularly reactive and volatile, they are extracted by procedures based on the transformation of PUAs into more stable products, such as ethyl ester by the Wittig reaction with carbetoxyethylidene-triphenylphosphorane (CET-TPP) [31], [32], or pentafluorobenzyl-oxime derivative by *O*-(2,3,4,5,6- pentafluorobenzyl) hydroxylamine hydrochloride (PFBHA·HCl) [24]. The quantification of particulate PUA concentration was performed following the method of d'Ippolito et al. [31] in March 2002 and 2004 and the method of Wichard et al. [24] in March 2005 and 2006. For the d'Ippolito et al. method, cells were collected by centrifugation (1200g, 10 minutes) and the pellet was frozen in liquid nitrogen and stored at −80°C until further analysis. PUAs were then analyzed according to [31]. For the Wichard et al. method, cells were concentrated on a 1.2 μm GF/C filter (Whatmann, Dassel, DE) under moderate vacuum (∼500 mbar). Cells were rinsed with 1 mL of 25 mM PFBHA (Roth, Karlsruhe, DE) solution in Tris-HCl 100 mM, pH 7.2. The cell suspension was then transferred to a 4 mL glass vial (Macherey-Nagel, Düren, DE) and five microlitres of internal standard (benzaldehyde, 1 mM in methanol, Sigma-Aldrich, DE) was added. The samples were incubated 1 h at room temperature and successively frozen in liquid nitrogen and stored at −80°C until further analysis. Concentrations of PUAs were then analyzed by gas chromatography–mass spectrometry following Wichard et al. method [24]. Particulate concentration of PUAs was calculated by dividing the amount of PUAs produced after mechanical cell disruption by the number of known-PUA producing diatom cells (mainly *Skeletonema marinoi*) [33].

The quantification of dissolved PUAs was performed in March 2006 following the method of Vidoudez and Pohnert [22]. One liter of surface seawater was gently filtered through 1–2 cm layer of dry commercial washed sea sand using a Büchner funnel with two layers of 25 μm Whatman filter paper. The filtered seawater, together with 5 μL of internal standard (1 mM Benzaldehyde in methanol), was run through a C18 cartridge column (Chromabond C18 ec, Machery-Nagel, Düren, DE) and the PUAs eluted by adding 6 ml of the derivatizing reagent (25 mM PFBHA in methanol). The samples were incubated 1 h at room temperature and then frozen in liquid nitrogen and stored at −80°C until further analysis. Concentrations of PUAs were then analyzed by gas chromatography–mass spectrometry following Wichard et al. method [24].

### Statistical Analyses

Lysis rates were compared with PUA concentrations using reduced major axis model II regression [34]. A permutation test was performed to determine the significance of the slopes and to calculate the Pearson coefficients of correlation. Statistical analyses were performed using the R computing environment (R Core Team, 2013), and judging significance at the 95% confidence level.

## Results

In all cruises, satellite-based chlorophyll *a* concentrations indicate that the highest phytoplankton biomass in the North Adriatic Sea occurred in the Po river plume along the western coastline of transects 2 and 3 (Fig. 1). The same was true for inorganic nutrients, with the highest concentrations observed in the Po river plume (Table 1). The mean of molar particulate organic C∶N ratios along transect 1 were close to the Redfield ratio (6.6) (6.89, 6.76, 6.79 for March 2004, 2005 and 2006, respectively) (Table 1). High C∶N ratios were observed along transect 2 in March 2002 and 2006 (11.45 and 9.92, respectively), which were associated with high POC concentrations (1155 and 403 μg L^−1^, respectively). The phytoplankton community was mainly composed of diatoms in March 2002, 2005 and 2006, representing 84, 75 and 78% of all counted cells (Table 1). The diatom *Skeletonema marinoi* dominated the diatom community numerically, with mean abundances of 4.06, 1.09 and 1.50×10^6^ cells L^−1^ representing 66, 89 and 87% of all diatom cells in March 2002, 2005 and 2006, respectively (Table 1), except along transects 1 and 2 in March 2006 where the diatom community was dominated by *Stephanodiscus* spp. and *Chaetoceros compressus*, respectively (Table 2). In March 2004, the phytoplankton assemblage was dominated by the prymnesiophyte *Emiliania huxleyi*, while diatom cell abundance was an order of magnitude lower than other years (Table 1 and 3).

**Table 1:** Nutrient, chlorophyll a concentrations, phytoplankton cell abundances and composition for each transect in the Northern Adriatic Sea.

Cruise and Transect		Si (μM)	DIN (μM)	Pi (μM)	POC (μg L^−1^)	C:N	Chl (μg L^−1^)	Phytoplankton (10^6^ cells L^−1^)			N
								*S. marinoi*	Other diatoms	Non-diatoms	
March	*1*	—	—	—	—	—	—	—	—	—	—
2002	*2*	11.05	27.45	0.19	1155	11.45	2.64	4.06	2.05	1.18	6
		0.36–59.09	2.24–126.70	0.04–0.85	397–2405	7.26–14.35	0.47–7.12	1.44–8.69	1.08–5.24	0.65–1.90	
(03/12)	*3*	—	—	—	—	—	—	—	—	—	—
	***mean***	**11.05**	**27.45**	**0.19**	**1155**	**11.45**	**2.64**	**4.06**	**2.05**	**1.18**	**6**
	***range***	**0.36–59.09**	**2.24–126.70**	**0.04–0.85**	**397–2405**	**7.26–14.35**	**0.47–7.12**	**1.44–8.69**	**1.08–5.24**	**0.65–1.90**	
March	*1*	3.72	9.54	0.09	125	6.89	0.22	0.00	0.02	0.66	5
2004		2.92–4.58	7.72–13.32	0.04–0.17	74–234	5.94–7.91	0.14–0.27	0.00–0.01	0.00–0.07	0.45–0.96	
	*2*	11.16	65.31	0.44	159	7.39	0.42	0.02	0.12	0.78	6
(02/26–		2.00–50.13	1.58–362.23	0.04–2.34	87–436	4.05–9.98	0.26–0.93	0.00–0.04	0.00–0.43	0.45–1.18	
03/03)	*3*	5.68	46.47	0.27	216	6.18	0.33	0.41	0.08	0.39	4
		3.02–8.40	4.40–159.29	0.05–0.83	115–316	5.11–7.26	0.29–0.38	0.02–0.81	0.04–0.08	0.39–1.26	
	***mean***	**7.22**	**41.69**	**0.28**	**155**	**7.00**	**0.33**	**0.05**	**0.07**	**0.73**	**15**
	***range***	**2.00–50.13**	**1.58–362.23**	**0.04–2.34**	**74–436**	**4.05–9.98**	**0.15–0.93**	**0.00–0.81**	**0.00–0.43**	**0.39–1.26**	
March	*1*	1.12	1.21	0.10	115	6.76	1.33	0.10	0.20	0.40	4
2005		0.10–2.23	0.73–1.62	0.04–0.26	72–202	5.46–7.59	0.79–1.70	0.01–0.17	0.01–0.35	0.11–0.42	
	*2*	1.02	2.92	0.08	92	7.02	1.69	0.34	0.33	0.54	8
(03/09–		0.10–2.61	0.51–11.24	0.04–0.12	73–107	3.60–9.25	0.46–2.19	0.00–0.66	0.02–2.36	0.13–1.99	
03/16)	*3*	2.78	10.39	0.10	185	6.05	3.60	4.22	0.16	0.20	3
		0.68–6.31	0.91–27.78	0.02–0.14	95–356	4.95–6.89	2.37–4.43	0.02–14.95	0.03–3.19	0.23–2.55	
	***mean***	**1.40**	**3.96**	**0.09**	**116**	**6.75**	**1.98**	**1.09**	**0.13**	**0.42**	**15**
	***range***	**0.10–6.31**	**0.51–27.78**	**0.02–0.26**	**72–356**	**3.60–9.25**	**0.46–4.43**	**0.00–14.95**	**0.01–3.19**	**0.11–2.55**	
March	*1*	2.91	2.80	0.04	120	6.79	2.61	0.00	0.21	0.6	5
2006		2.07–5.21	0.58–9.18	0.01–0.09	83–164	5.94–7.73	1.21–7.11	0.00–0.62	0.00–0.32	0.16–0.36	
	*2*	8.84	20.42	0.27	403	9.92	5.83	0.11	0.41	0.34	8
(03/17–		1.91–53.56	0.85–147.99	0.03–1.73	84–865	7.19–12.42	0.98–8.12	0.00–1.00	0.00–0.65	0.00–0.78	
03/20)	*3*	3.53	8.13	0.05	199	6.23	5.82	12.89	0.31	2.80	4
		1.58–7.05	2.52–16.54	0.03–0.10	95–336	4.95–7.20	3.14–7.45	0.14–21.48	0.02–0.48	0.35–3.79	
	***mean***	**5.99**	**12.61**	**0.16**	**272**	**8.13**	**5.01**	**1.50**	**0.23**	**0.49**	**17**
	***range***	**1.58–53.56**	**0.58–147.99**	**0.01–1.73**	**83–865**	**4.95–12.43**	**0.98–8.12**	**0.00–21.48**	**0.00–0.65**	**0.00–1.79**	

Mean and range of silicic acid (Si, μM), dissolved inorganic nitrogen (DIN, μM), phosphate concentrations (Pi, μM), particulate organic carbon (POC, μg L^−1^), molar particulate C:N ratio (mol:mol), chlorophyll *a* concentrations (μg L^−1^, based on fluorometric detection after HPLC separation), *Skeletonema marinoi* (*S. marinoi*), other diatoms and non-diatom cell abundances (10^6^ cells L^−1^) and number of stations (N) sampled per transect in March 2002, 2004, 2005 and 2006 in the Northern Adriatic Sea.

**Table 2:** Phytoplankton species composition and cell abundances for each transect in the Northern Adriatic Sea.

Species	Cell abundances (10^3^ cells L^−1^)															
	March 2002				March 2004				March 2005				March 2006			
	1	2	3	Total	1	2	3	Total	1	2	3	Total	1	2	3	Total
**Bacillariophyceae**	—	**6118**	—	**6118**	**27**	**139**	**498**	**115**	**305**	**673**	**4377**	**1221**	**208**	**514**	**13194**	**1737**
*Chaetoceros affinis*	—	—	—	—	0	0	0	0	46	17	9	25	0	0	147	16
*Chaetoceros calcitrans*	—	—	—	—	10	2	9	7	1	34	0	15	0	0	0	0
*Chaetoceros compressus*	—	—	—	—	0	0	0	0	0	14	6	7	0	392	0	144
*Chaetoceros* spp.	—	—	—	—	2	2	13	3	88	172	84	209	27	0	0	6
*Leptocylindrus danicus*	—	—	—	—	0	0	0	0	30	35	30	32	0	0	0	0
*Navicula* spp.	—	—	—	—	0	7	0	2	3	4	1	3	21	1	0	5
*Pseudonitzschia delicatissima*	—	—	—	—	0	0	0	0	1	2	0	1	0	0	98	11
*Skeletonema marinoi*	—	4065	—	4065	3	16	414	47	105	339	4219	1087	1	108	12889	1504
*Stephanodiscus* spp.	—	—	—	—	0	0	0	0	0	0	0	0	125	0	0	28
*Thalassiosira* spp.	—	—	—	—	0	3	17	3	2	28	4	14	0	0	0	0
Unidentified	—	—	—	—	3	51	26	24	0	0	2	1	0	0	0	0
**Dinophyceae**	—	**39**	—	**39**	**20**	**13**	**20**	**17**	**16**	**15**	**9**	**14**	**1**	**20**	**50**	**19**
*Alexandrium minutum*	—	—	—	—	0	0	0	0	0	0	0	0	0	0	49	5
*Prorocentrum minimum*	—	—	—	—	3	3	0	3	2	2	2	2	0	19	0	13
Unidentified	—	—	—	—	10	8	9	10	8	7	5	7	0	0	0	0
**Prymnesiophyceae**	—	**14**	—	**14**	**158**	**125**	**35**	**157**	**26**	**15**	**13**	**19**	**1**	**80**	**98**	**65**
*Emiliania huxleyi*	—	—	—	—	156	124	35	156	25	15	10	17	1	79	98	64
**Cryptophyceae**	—	**40**	—	**40**	**100**	**95**	**99**	**111**	**40**	**89**	**49**	**63**	**1**	**1**	**343**	**39**
**Prasinophyceae**	—	**0**	—	**0**	**8**	**4**	**4**	**6**	**0**	**0**	**0**	**0**	**0**	**0**	**0**	**0**
**Other flagellates**	—	**1081**	—	**1081**	**377**	**539**	**242**	**441**	**317**	**417**	**125**	**319**	**256**	**318**	**2401**	**536**
**Total**	—	**7294**	—	**7294**	**689**	**920**	**886**	**849**	**704**	**1210**	**4573**	**1636**	**466**	**853**	**15989**	**2231**

Species composition and mean of phytoplankton cell abundances (10^3^ cells L^−1^) per transect in March 2002, 2004, 2005 and 2006 in the Northern Adriatic Sea. Species are shown only if their mean per transect were once higher than 5×10^3^ cells L^−1^. Values are the total of all counted cells for the group, including the species not presented here. In March 2002, species were identified at the class level, except for the diatom *S. marinoi*.

**Figure 2:**
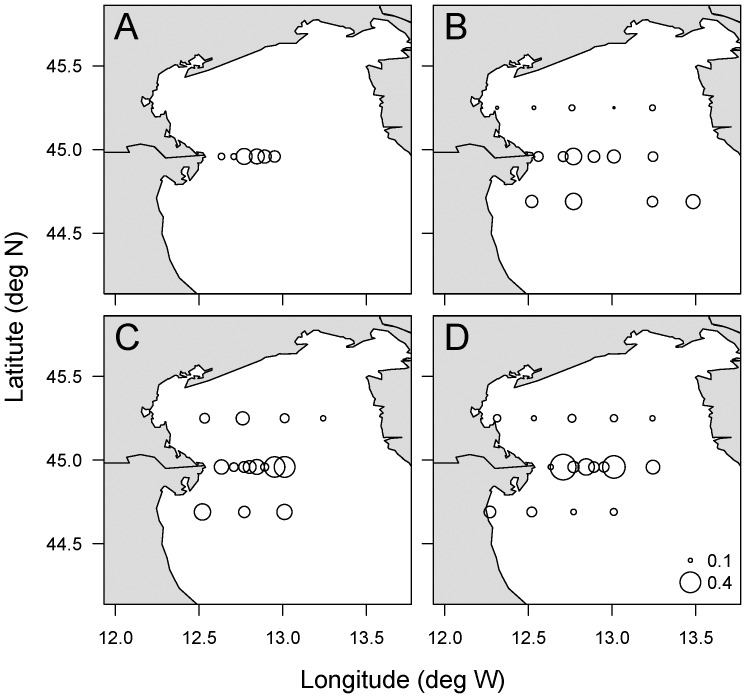
Lysis rates in the North Adriatic Sea. Lysis rate (d^−1^), calculated using the esterase method [29] in March a) 2002, b) 2004, c) 2005 and d) 2006. The diameter of the symbols is proportional to the measurement.

**Figure 3:**
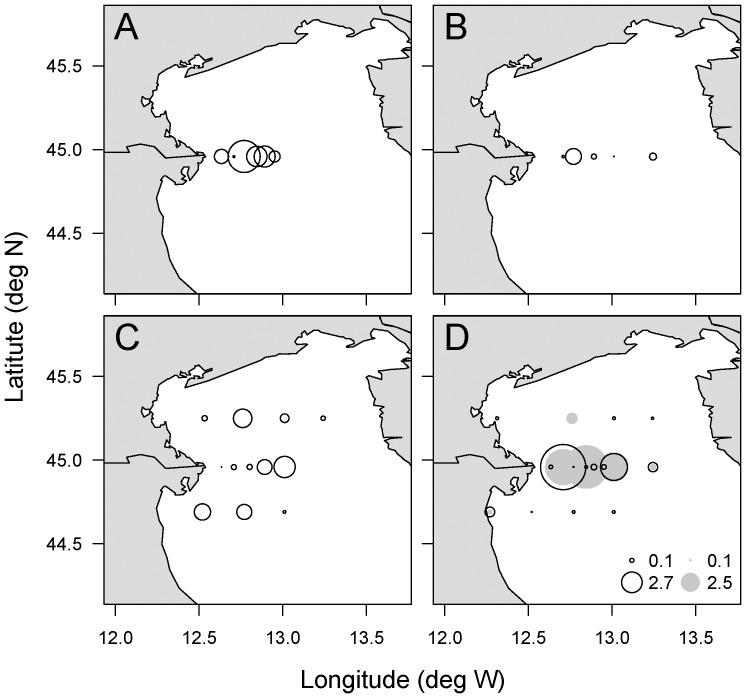
Relationship between lysis rates and concentrations of particulate or dissolved PUAs). a) Lysis rates and concentration of particulate PUAs (particulate PUAs, fmol cell^−1^) measured a) in March 2002–2004 (open triangles), and in March 2005–2006 (black circles); or c) during the time course experiment in March 2005. Lysis rates and concentrations of dissolved PUAs (dissolved PUAs, nM) measured b) in March 2006 and d) during the time course experiment in March 2006. Dashed lines represent model II linear regression of plotted data and R represents Pearson coefficient of correlation. Grey and black solid lines in panel a) represent model II linear regression of 2002–2004 and 2005–2006 data.

Lysis rates ranged from 0.03 to 0.38 d^−1^, with an average of 0.16±0.01 d^−1^ (Fig. 2). Transect 1 showed lower mean lysis rate values (0.06, 0.13, 0.09 d^−1^, in March 2004, 2005 and 2006, respectively) than those obtained in transect 2 (0.17, 0.17, 0.21, 0.21 d^−1^, in March 2002, 2004, 2005 and 2006, respectively) or transect 3 (0.20, 0.21, 0.13 d^−1^, in March 2004, 2005 and 2006, respectively) (Fig. 2 and Table 1, ANOVA, P<0.05). Concentrations of total particulate PUAs were highly variable with values ranging from undetectable amount to 5.37 fmol cell^−1^, with a mean of 1.16 fmol cell^−1^ (Table 3). Particulate heptadienal represented, on average, 68% of the total PUAs while octadienal and octatrienal represented only 18% and 13%, respectively (Table 3). Compositions of particulate PUAs changed in March 2006 along transect 2, with octadienal and heptadienal representing 79% and 16% of the total PUAs (Table 3). Concentrations of total particulate PUAs in March 2004 (0.74 fmol cell^−1^) was significantly lower than March 2002 (1.76 fmol cell^−1^), 2005 (1.09 fmol cell^−1^) and 2006 (0.96 fmol cell^−1^) (Tukey Multiple Comparison post-test, P<0.05) (Table 3). The highest concentration of particulate PUAs (5.37 fmol cell^−1^, composed exclusively of octadienal) was observed in March 2006 along transect 2 near the Po river mouth, where the diatom community was dominated by *Chaetoceros compressus* (76%) (Table 2) and where low concentrations of inorganic nitrogen and phosphate were recorded (<1.6 µM and <0.01 µM, respectively). The concentrations of total particulate PUAs were positively correlated with lysis rates (R = 0.69, P<0.001) (Fig. 3A), and not with chlorophyll *a* concentrations, nutrient concentrations or cell abundances of *S. marinoi* (P>0.1 in all cases) (data not shown).

**Table 3:** Esterase activity, lysis rate, concentrations of particulate and dissolved polyunsaturated aldehydes for each transect in the Northern Adriatic Sea.

Cruise and Transect		NEH (nM h^−1^)	DEA (nM h^−1^)	PEA (nM h^−1^)	LR^1^ (d^−1^)	Particulate PUA concentration (fmol cell^−1^)				Dissolved PUA concentration (nM)			
						7:2	8:2	8:3	Total	7:2	8:2	8:3	Total
March	*1*	—	—	—	—	—	—	—		—	—	—	—
2002	*2*	—	5.20	13.00	0.17	1.21	0.10	0.45	1.76	—	—	—	—
			3.60–8.51	5.21–33.00	0.09–0.24	0.18–1.75	0.00–0.22	0.02–1.20	0.23–2.51				
(03/12)	*3*	—	—	—	—	—	—	—	—	—	—	—	—
	***mean***	—	**5.20**	**13.00**	**0.17**	**1.21**	**0.10**	**0.45**	**1.76**	—	—	—	—
	***range***		**3.60–8.51**	**5.21–33.00**	**0.09–0.24**	**0.18–1.75**	**0.00–0.22**	**0.02–1.20**	**0.23–2.51**				
March	*1*	10.87	7.93	46.83	0.06	—	—	—	—	—	—	—	—
2004		7.98–15.44	4.73–10.41	15.99–76.89	0.03–0.09								
	*2*	13.68	9.81	18.30	0.17	0.42	0.16	0.16	0.74	—	—	—	—
(02/26–		11.23–15.96	3.35–20.77	5.08–42.69	0.14–0.24	0.04–0.96	0.00–0.66	0.04–0.40	0.08–1.89				
03/03)	*3*	14.79	19.11	27.74	0.20	—	—	—	—	—	—	—	—
		12.11–17.07	11.27–31.60	17.03–38.12	0.16–0.24								
	***mean***	**13.04**	**11.66**	**30.33**	**0.14**	**0.42**	**0.16**	**0.16**	**0.74**	—	—	—	—
	***range***	**7.97–17.07**	**3.35–31.60**	**5.08–76.89**	**0.03–0.24**	**0.04–0.96**	**0.00–0.66**	**0.04–0.40**	**0.08–1.89**				
March	*1*	12.38	7.23	18.24	0.13	1.00	0.01	0.05	1.06	—	—	—	—
2005		11.84–13.12	3.55–9.65	13.54–24.89	0.08–0.20	0.53–1.83	0.00–0.05	0.00–0.20	0.52–2.08				
	*2*	13.52	9.30	16.68	0.21	1.01	0.02	0.05	1.08	—	—	—	—
(03/09–		3.16–19.20	5.53–17.70	6.14–31.77	0.12–0.31	0.05–2.54	0.00–0.07	0.00–0.08	0.06–2.54				
03/16)	*3*	9.96	15.08	24.73	0.21	0.81	0.15	0.16	1.12	—	—	—	—
		7.69–13.30	12.67–18.14	19.20–29.43	0.17–0.24	0.00–0.45	0.00–0.31	0.00–0.30	0.00–1.91				
	***mean***	**12.51**	**9.90**	**18.70**	**0.19**	**0.96**	**0.05**	**0.08**	**1.09**	—	—	—	—
	***range***	**3.16–19.20**	**3.55–18.14**	**6.14–31.77**	**0.08–0.31**	**0.00–2.54**	**0.00–0.45**	**0.00–0.30**	**0.00–2.54**				
March	*1*	11.81	2.95	10.01	0.09	0.18	0.03	0.00	0.21	0.19	0.19	0.23	0.61
2006		8.76–13.16	0.72–4.82	2.22–14.63	0.07–0.12	0.00–0.28	0.00–0.09	0.00–0.00	0.00–0.35				
	*2*	13.01	5.65	8.40	0.21	0.23	1.13	0.07*	1.43	0.04	0.84	0.21*	1.09
(03/17–		10.26–19.23	1.53–17.77	2.76–17.41	0.08–0.38	0.00–1.12	0.00–5.37	0.00–0.56*	0.00–5.37	0.00–0.18	0.00–2.40	0.00–0.80*	0.06–2.53
03/20)	*3*	14.54	3.09	7.38	0.13	0.30	0.11	0.00	0.42	0.05	0.08	0.03*	0.17
		10.88–17.69	1.09–6.92	4.32–13.14	0.08–0.17	0.00–0.85	0.00–0.32	0.00–0.00	0.00–1.17	0.00–0.16	0.03–0.12	0.02–0.05*	0.13–0.22
	***mean***	**13.04**	**4.25**	**8.63**	**0.16**	**0.24**	**0.68**	**0.04**	**0.96**	**0.05**	**0.57**	**0.16**	**0.79**
	***range***	**8.76–19.23**	**0.72–17.77**	**2.22–17.41**	**0.07–0.38**	**0.00–1.12**	**0.00–5.37**	**0.00–0.56**	**0.00–5.37**	**0.00–0.19**	**0.00–2.40**	**0.00–0.80**	**0.06–2.53**

1 Lysis rate calculated according to Riegman & Winter 2003.

Mean and range of percent of non-enzymatic hydrolysis (NEH, nM h^−1^) of fluorescein to esterase activity, particulate and dissolved esterase activity (PEA and DEA, nM h^−1^, both corrected from NEH), lysis rate (LR, d^−1^) and concentrations of particulate (fmol cell^−1^) and dissolved (nM) polyunsaturated aldehyde (PUAs) (7:2, 8:2 and 8:3 for heptadienal, octadienal and octatrienal, respectively) per transect in March 2002, 2004, 2005 and 2006 in the Northern Adriatic Sea. * Traces of decatrienal were also detected.

**Figure 4:**
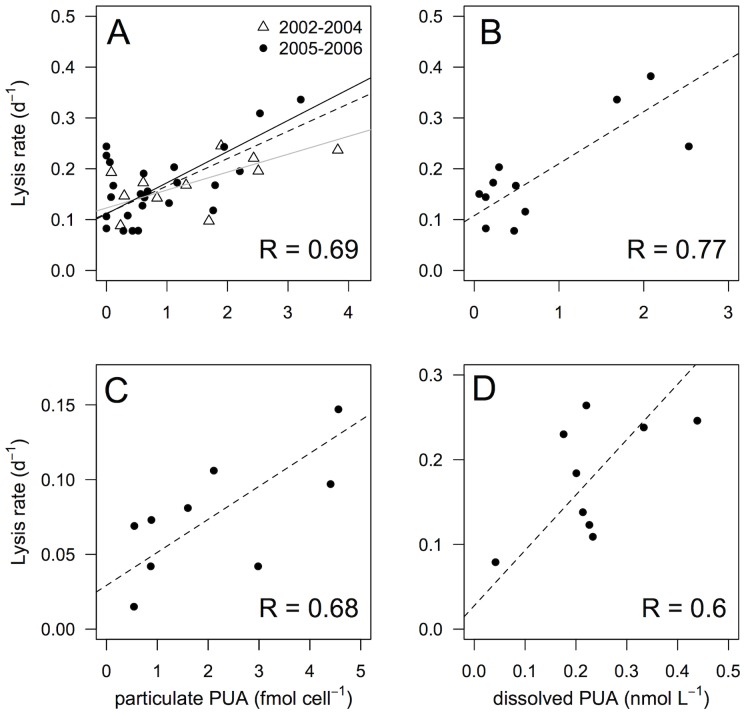
Concentrations of total particulate and dissolved PUAs in the North Adriatic Sea. Concentrations of total particulate PUAs (fmol cell^−1^, black circles) were measured using [31] in March a) 2002, and using [24]in March b) 2004, c) 2005 and d) 2006 and concentrations of dissolved PUAs (nM, grey circles) were measured using [26] in March 2006. The diameter of the symbols is proportional to the measurement.

Dissolved PUAs were detected at each station in March 2006, with concentrations ranging from 0.06 to 2.53 nM (Fig. 4d and Table 3). Consistent with particulate PUAs, dissolved PUAs were mostly composed of octadienal (Table 3). The highest concentrations of dissolved PUAs were observed along transect 2 (1.09 nM), located at the Po river mouth, and coincided with high lysis rates (0.21 d^−1^) and high concentration of particulate PUAs (1.43 fmol cell^−1^). A good correlation was observed between the concentrations of total dissolved PUAs and lysis rates (R = 0.77, P = 0.005) (Fig. 3B). In general, higher concentrations of dissolved PUAs were associated with periods when *S. marinoi* dominated the phytoplankton community, though the highest concentration of dissolved octadienal (2.40 nM) was measured when cell lysis was high (0.24 d^−1^) but neither *S. marinoi* cells nor particulate PUAs were detected. No correlation was observed between concentrations of total dissolved and particulate PUAs (P>0.1) (data not shown).

**Figure 5:**
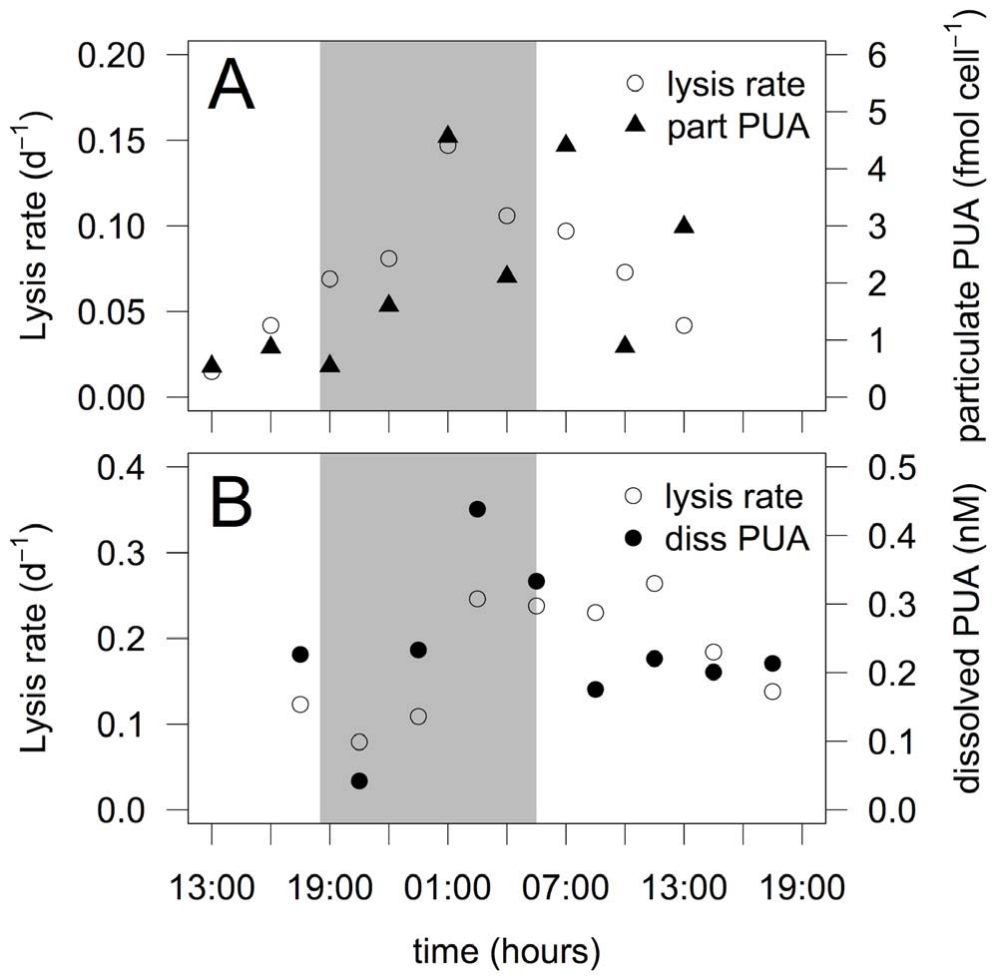
Lysis rate and concentrations of particulate and dissolved PUA concentrations observed during a diatom blooms over a 27-h period. a) Lysis rate (d^−1^) and concentrations of particulate PUAs (particulate PUAs, fmol cell^−1^) measured in March 2005. b) Lysis rate (d^−1^) and concentrations of dissolved PUAs (dissolved PUAs, nM) in March 2006.

A 27 h-time course experiment was performed in March 2005 and 2006 at a station near shore along the transect 3 (Fig. 1) where *S. marinoi* cell abundance (1.8×10^7^ cell L^−1^ and 2.2×10^7^ cell L^−1^, respectively) represented 98% and 97% of diatom cells. Lysis rates varied from 0.02 to 0.15 d^−1^ in March 2005 and from 0.08 to 0.26 d^−1^ in March 2006 (Fig. 5A and 5B) over the diel cycle, driven by changes in dissolved esterase activity (Fig. S1A and S1B). No significant differences in lysis rates were observed between dark and light periods in either experiment (Student's *t*-test, P>0.1 and P>0.9 for 2005 and 2006, respectively), though the statistical tests were of low power because there we relatively few data points. Particulate and dissolved PUAs were composed of 70% and 66% octadienal, respectively, with concentrations ranging over almost an order of magnitude (from 0.54 to 4.56 fmol cell^−1^ and 0.04 to 0.44 nM for concentrations of particulate and dissolved PUAs, respectively) (Fig. 5A and 5B). Concentrations of total particulate and dissolved PUAs were positively correlated with lysis rates (R = 0.69, P = 0.02 and R = 0.60, P = 0.04, respectively) (Fig 4C and 4D). No significant differences related to the time of day were found for particulate and dissolved concentrations of PUAs (Student's *t*-test, P>0.05 and P>0.5, respectively).

## Discussion

Our results indicate that high phytoplankton lysis rates (up to 0.38 d^−1^) can occur during diatom blooms in the North Adriatic, which is in good agreement with previous phytoplankton loss rate estimates based on ^14^C uptake [35]. These rates are very high, since phytoplankton net growth rates in the region range from 0.1 to 0.5 d^−1^ in spring [35], [36]. Although esterase assays are prone to overestimation of phytoplankton lysis rates (because a small but significant fraction of both DEA and PEA can originate from heterotrophs, rather than phytoplankton[37]), our results support the idea that phytoplankton cell lysis is an important loss factor driving the dynamics of phytoplankton dynamics. This has also been noted for individual species, for example, winter blooms of the prymnesiophyte *Phaeocystis* spp. in the North Sea [29], [30], [38].

Average cell lysis rates were three times higher during the diatom blooms in the Adriatic Sea (0.16 d^−1^) than those measured during non-diatom blooms in the North Sea (0.05 d^−1^) [30], [38]. Numerous factors could be responsible for this difference. Several studies have shown that diatom cell lysis can be triggered by nutrient depletion [39]–[41] or exposure to high light [42], conditions which existed during our studies. However, no significant correlations were found between lysis rates and the levels of inorganic nutrients, POC, molar particulate C∶N ratio or light exposure during the survey. Field studies have shown that diatom cell lysis can also be exogenously triggered by biotic factors, such as viruses [43], and algicidal bacteria [45], [46]. In addition, there is growing evidence that the production of algae-derived toxic compounds, such as PUAs, could play a significant role in determining algal bloom dynamics and fate [47], [48]. However, direct links between environmental factors and phytoplankton cell lysis rates are very difficult to demonstrate in nature; these factors are dynamic and they can interact with each other [49]. Moreover, Brussaard & Riegman [49] showed that the magnitude of cell lysis induced by nutrient limitation can be modified by the presence of bacteria.

Concentrations of total particulate PUAs varied widely across locations (from 0 to 5.37 fmol cell^−1^) but were remarkably similar among the four cruises despite significant changes in diatom compositions and environmental conditions (Table 1 and 2). At stations where *S. marinoi* dominated the diatom community, particulate PUAs were mainly composed of heptadienal, and, to a minor extent, octadienal and octatrienal (Table 3), in good agreement with field observations in the region [50]. In March 2006, particulate PUAs along transect 2 were mainly composed of octadienal, with trace of decatrienal, which may be due to the high numbers of *Chaetoceros compressus* (Table 2), a known octadienal and decatrienal-producing species [33].

Total dissolved PUAs ranged from 0 to 2.53 nM (Table 3), which is about 2 orders of magnitude lower than concentrations measured in S. marinoi cultures (>200 nM ) [22] but in good agreement with previous *in situ* measurements in the region [50]. The highest concentrations of dissolved PUAs were measured when cell lysis was high (0.24 d^−1^) but at these times, no particulate PUAs were detected. This result is consistent with previous work during *S. marinoi* blooms showing that traces of dissolved heptadienal and octadienal were found at several stations where no particulate PUAs were detected [26]. In the latter study, one possible explanation is that not all PUA-producing organisms were collected; samples for particulate PUAs were collected using GF/C filters (nominal pore size 1.2 µm). Thus smaller cells such as picoplankton and bacteria were under-sampled, and both groups have been reported to contain small amounts of particulate heptadienal [26]. An alternative explanation is that, at the point of sampling, all PUA producing species had already lysed, leaving dissolved PUAs as the only pool. Finally, preliminary results show that lipoxygenases, key enzymes for PUA production, are still active 48 h after being released in seawater [51], suggesting that free enzymes could still produce PUAs after cells have disappeared.

Positive correlations between both concentrations of particulate and dissolved PUAs and lysis rates along transects and over the 27-h time course experiments (Fig. 3) suggest a tight coupling between the three variables. We hypothesize that the positive correlation between concentrations of particulate PUAs and lysis rates results because particulate PUAs in diatoms increase under physiological stress, as observed in cultures [20], [25], and diatom cell lysis similarly occurs in response to stress (e.g., nutrient limitation). Together with the fact that the composition of particulate and dissolved PUAs is very similar, the correlation between dissolved PUAs and lysis rates support the hypothesis that PUAs are released in seawater following cell lysis. An alternative hypothesis is that grazing results in dissolved PUA in the seawater. However, micro-scale examination has failed to detect production of dissolved PUAs around the mouths of grazing copepods [52].

Dissolved PUA concentrations and cell lysis are also correlated, raising the question of whether concentrations of dissolved PUAs are high enough to induce phytoplankton cell lysis. Although diatom cell growth can be inhibited by nanomolar PUA concentrations in culture [25], cell lysis has been shown to occur only when cells are exposed to micromolar PUA concentrations [4], [9], [10]. The concentrations of dissolved PUAs during the survey would therefore be at least one order of magnitude lower than the toxic levels established in cultures, suggesting that PUA concentrations were probably not high enough to induce cell lysis. However, bulk measurements of PUAs in seawater may not reflect the local concentrations in the vicinity of the PUA-releasing cells. Based on a simple diffusion model, micromolar concentrations of PUAs would be found at 1 µm-distance from a single *S. marinoi* cells (see [10] for more details). Methods for the quantification of microscale chemical gradients, such as the surface plasmon resonance detection method coupled with antibodies [53], will be needed to determine the ecological consequences of PUAs on the structure and dynamics of diatom populations.

## Supporting Information

Figure S1 **Lysis rate and dissolved esterase activity measured during a diatom blooms over a 27-h period.** Lysis rate (d^-1^) and dissolved esterase activity (EA, nM h^-1^) measured a) in March 2005 and b) March 2006.
(TIFF)
