# Correction: Phytoplankton Cell Lysis Associated with Polyunsaturated Aldehyde Release in the Northern Adriatic Sea

**DOI:** 10.1371/journal.pone.0098727

**Published:** 2014-05-27

**Authors:** The PLOS ONE Staff

There is an error in affiliation 1 for authors François Ribalet, Adrianna Ianora, Antonio Miralto, Giovanna Romano, and Raffaella Casotti. Affiliation 1 should be: Stazione Zoologica Anton Dohrn, Napoli, Italy.

The legends for Figure 3 and Figure 4 are swapped. Please see the complete, corrected Figure 3 here.

**Figure 3:**
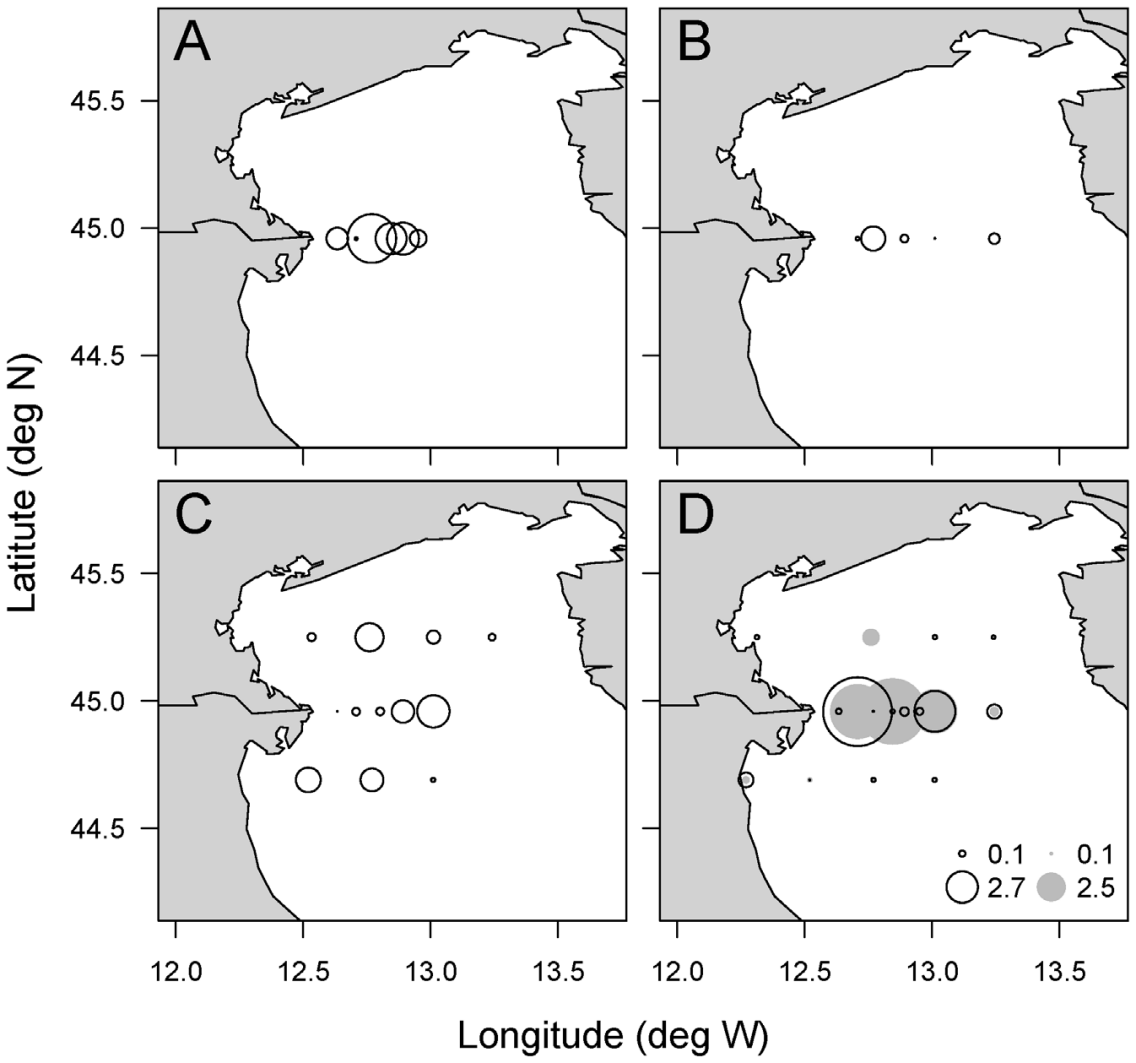
Concentrations of total particulate and dissolved PUAs in the North Adriatic Sea. Concentrations of total particulate PUAs (fmol cell−1, black circles) were measured using [31] in March a) 2002, and using [24]in March b) 2004, c) 2005 and d) 2006 and concentrations of dissolved PUAs (nM, grey circles) were measured using [26] in March 2006. The diameter of the symbols is proportional to the measurement.

Please see the complete, corrected Figure 4 here.

**Figure 4:**
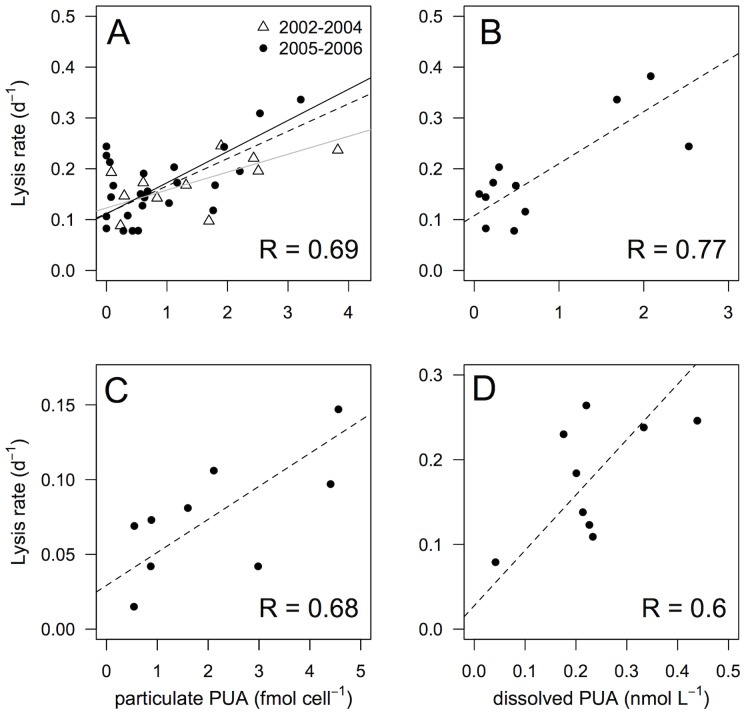
Relationship between lysis rates and concentrations of particulate or dissolved PUAs). a) Lysis rates and concentration of particulate PUAs (particulate PUAs, fmol cell−1) measured a) in March 2002–2004 (open triangles), and in March 2005–2006 (black circles); or c) during the time course experiment in March 2005. Lysis rates and concentrations of dissolved PUAs (dissolved PUAs, nM) measured b) in March 2006 and d) during the time course experiment in March 2006. Dashed lines represent model II linear regression of plotted data and R represents Pearson coefficient of correlation. Grey and black solid lines in panel a) represent model II linear regression of 2002–2004 and 2005–2006 data.
