# Supplementary figures and images for: Phytoplankton Cell Lysis Associated with Polyunsaturated Aldehyde Release in the Northern Adriatic Sea
Source: PLoS One. 2014 Jan 31;9(1):e85947. doi: 10.1371/journal.pone.0085947 (PMC3908894; doi:10.1371/journal.pone.0085947)

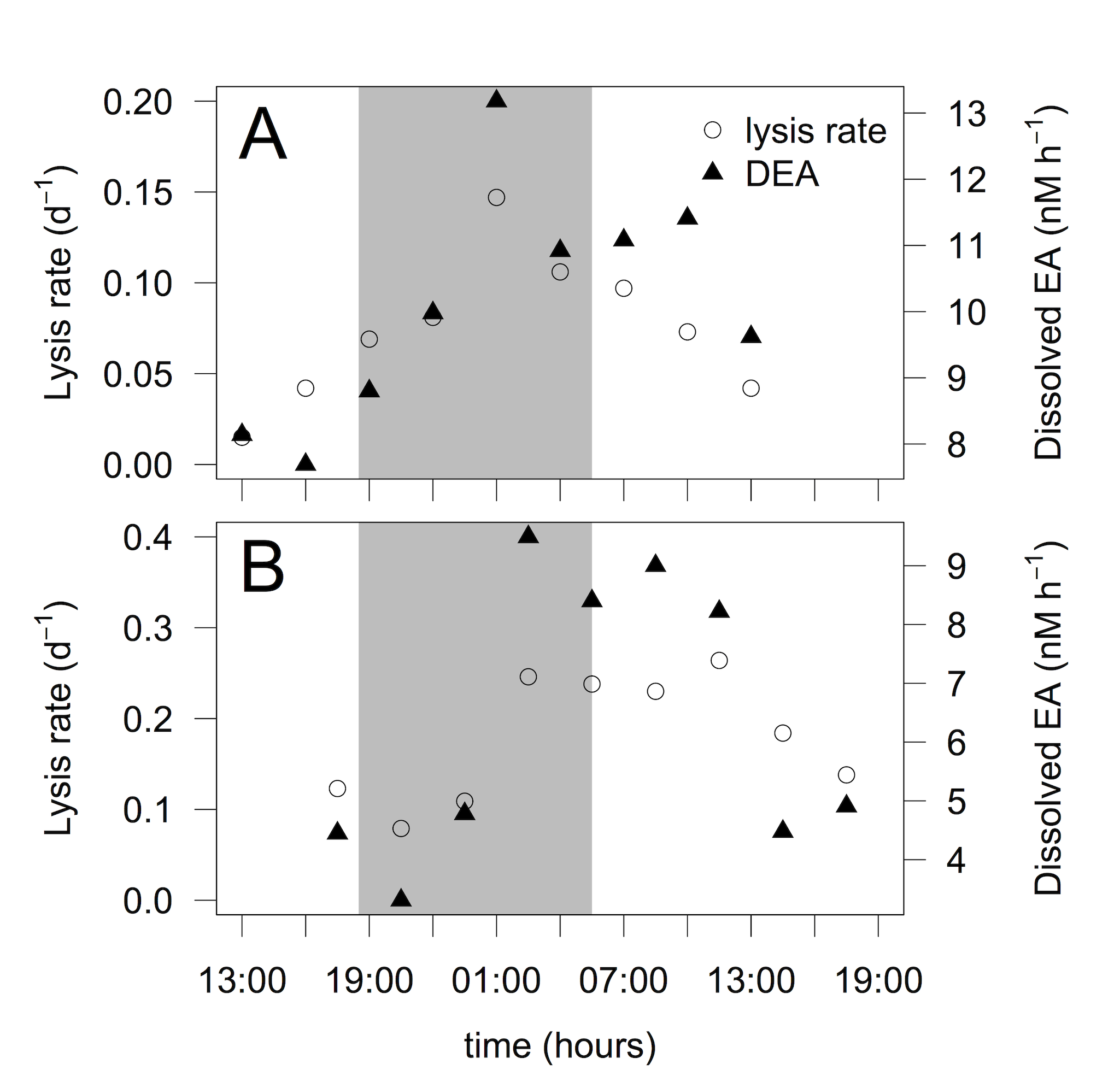

Supplement: Figure S1 — Lysis rate and dissolved esterase activity measured during a diatom blooms over a 27-h period. Lysis rate (d-1) and dissolved esterase activity (EA, nM h-1) measured a) in March 2005 and b) March 2006. (TIFF) [file pone.0085947.s001.tiff]
